# The Buffalo Medical Library Association

**Published:** 1883-01

**Authors:** A. H. Briggs

**Affiliations:** Secretary


### THE BUFFALO MEDICAL LIBRARY ASSOCIATION.

*First Meeting, December 15th, 1882.*

Present—Drs. Burwell, Hauenstein, Mann, Granger, Lothrop, W. W. Potter, Gay, Frederick, Brecht, Abbott, Hopkins, William Ring, Howe, Cary and Briggs.

On motion, Dr. George N. Burwell was elected Chairman and Dr. A. H. Briggs, Secretary.

Articles of incorporation, in accordance with the law of the State of New York, were adopted, and signed as incorporators by all present, and their signatures acknowledged before a Notary Public.

The following officers of the Association were then elected by ballot: President, George M. Burwell, M. D.; Vice-President, John Hauenstein, M. D.; Secretary, Albert H. Briggs, M. D.; Treasurer, C. C. Frederick, M. D.; Librarian, M. D. Mann, M. D.; Trustees, Drs. W. D. Granger, Henry R. Hopkins, Lucien Howe and Thomas Lothrop.

The following physicians were then elected incorporate members of the Association: Drs. Cary, Tremaine, Mackay, Hauenstein, Stockton, Granger, Packwood, O'Brien, Keene, Brown, Armstrong, Pryor, Hartwig, Hebenstreit, Grove, Johnson, Hopkins, Briggs, Lothrop, Burwell, Brecht, Potter, Abbott, Howe, Mynter, Slacer, Peterson, Long, Andrews, Cronyn, Rochester, Gay, Daggett, Mann, Fowler, Frederick, Samo, Pettitt, Chas. Ring, Putnam, Diehl, Daniels, Jewett, Folwell, Van Peyma, Bartow, Wyckoff, Miner, Tobie, Mixer and Davidson.

A constitution for the government of the Association was then adopted.

Adjourned. A. H. Briggs, *Secretary.*

